# Bioinoculants—Natural Biological Resources for Sustainable Plant Production

**DOI:** 10.3390/microorganisms10010051

**Published:** 2021-12-27

**Authors:** Sagar Maitra, Marian Brestic, Preetha Bhadra, Tanmoy Shankar, Subhashisa Praharaj, Jnana Bharati Palai, M. Mostafizur Rahman Shah, Viliam Barek, Peter Ondrisik, Milan Skalický, Akbar Hossain

**Affiliations:** 1 Department of Agronomy, M.S. Swaminathan School of Agriculture, Centurion University of Technology and Management, Paralakheundi 761 211, India; sagar.maitra@cutm.ac.in (S.M.); tanmoy@cutm.ac.in (T.S.); subhashisa.praharaj@cutm.ac.in (S.P.); jnana@cutm.ac.in (J.B.P.); 2 Department of Plant Physiology, Slovak University of Agriculture, Tr. A. Hlinku 2, 949 01 Nitra, Slovakia; peter.ondrisik@uniag.sk; 3 Department of Botany and Plant Physiology, Faculty of Agrobiology, Food, and Natural Resources, Czech University of Life Sciences Prague, Kamycka 129, 165 00 Prague, Czech Republic; skalicky@af.czu.cz; 4 Department of Biotechnology, M.S. Swaminathan School of Agriculture, Centurion University of Technology and Management, Paralakheundi 761 211, India; preetha.bhadra@cutm.ac.in; 5 Bangladesh Wheat and Maize Research Institute, Dinajpur 5200, Bangladesh; mostafiz.wrc@gmail.com; 6 Department of Water Resources and Environmental Engineering, Faculty of Horticulture and Landscape Engineering, Slovak University of Agriculture, Tr. A. Hlinku 2, 949 01 Nitra, Slovakia; viliam.barek@uniag.sk

**Keywords:** negative impact, green revolution, bioinoculants, climate change mitigation, agricultural sustainability

## Abstract

Agricultural sustainability is of foremost importance for maintaining high food production. Irresponsible resource use not only negatively affects agroecology, but also reduces the economic profitability of the production system. Among different resources, soil is one of the most vital resources of agriculture. Soil fertility is the key to achieve high crop productivity. Maintaining soil fertility and soil health requires conscious management effort to avoid excessive nutrient loss, sustain organic carbon content, and minimize soil contamination. Though the use of chemical fertilizers have successfully improved crop production, its integration with organic manures and other bioinoculants helps in improving nutrient use efficiency, improves soil health and to some extent ameliorates some of the constraints associated with excessive fertilizer application. In addition to nutrient supplementation, bioinoculants have other beneficial effects such as plant growth-promoting activity, nutrient mobilization and solubilization, soil decontamination and/or detoxification, etc. During the present time, high energy based chemical inputs also caused havoc to agriculture because of the ill effects of global warming and climate change. Under the consequences of climate change, the use of bioinputs may be considered as a suitable mitigation option. Bioinoculants, as a concept, is not something new to agricultural science, however; it is one of the areas where consistent innovations have been made. Understanding the role of bioinoculants, the scope of their use, and analysing their performance in various environments are key to the successful adaptation of this technology in agriculture.

## 1. Introduction

**Figure 1:**
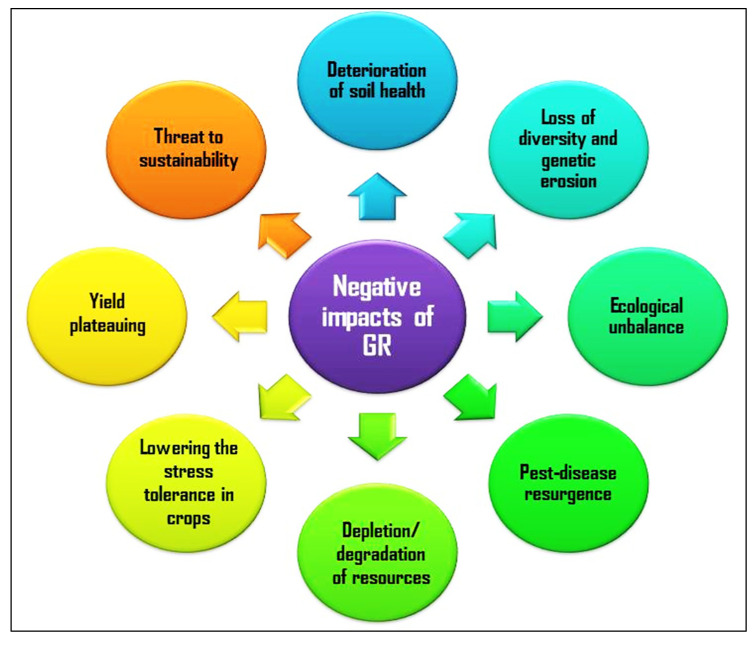
The negative impact of the Green Revolution on agriculture.

The first and foremost task of world agriculture is to produce enough food to fulfil the need of the future global human population, and the projection indicated that it could reach 9.7 and 11.2 billion in 2050 and 2100, respectively [1]. Another important challenge is to provide the raw material for some industries and animal feed for a huge livestock population. There was a significant increase in agricultural production in the recent past and between the period from 1960 and 2015; farm output was increased by three times [2]. Undoubtedly, green revolution technologies (GRTs) contributed a lot at the beginning of the journey started six decades back towards production enhancement in agriculture. Simultaneously, industrialization and urbanization also flourished which made a lengthy food supply chain with processing and value addition. Further, food demand was increased due to income growth and change in food habits and choices. Modern agriculture facilitated mechanization, monoculture, cultivation of improved varieties and hybrids of crops, use of chemical inputs directed to a generalization of the interacting mechanisms in agricultural systems and ultimately caused genetic erosion, greater susceptibility to abiotic stress and thus vulnerability in the cropping system. There was assured enhancement of production and productivity by the adoption of GRTs, but simultaneously brought uncertainty in farming with a threat to agricultural sustainability [3]. The Green Revolution (GR) was characterized by mainly research development and initiatives for the transfer of technology which increased agricultural productions worldwide, particularly in developing countries. The production enhancement noted was mainly due to the arrangement of assured irrigation, the inclusion of a huge quantity of chemical nutrients and the cultivation of ideotypes and high yielding varieties of crops. All these factors created tremendous pressure on the agricultural production system which already started to face multiple problems with shrinking land and deteriorating water resources [4]. The major negative impacts of GR observed were degradation of soil physicochemical and biological health, biodiversity loss and genetic erosion, ecological unbalance, pest-disease resurgence, depletion/degradation of resources including land, water and fossil fuel, lowering the stress tolerance in crops, yield plateauing and ultimately threat to sustainability [5] (Figure 1). 

During that period GRTs became the economically effective path to move. Soon after the realization of environmental issues, ideas were developed for the efficient use of available resources for sustaining farm output [6]. On the other hand, environmental problems were a relevant topic of discussion at different forums since the 1960s and the concept of ‘sustainable’ development was come into consideration [7]. Later, in 2000, the Millennium Development Goals and UN Sustainable Development Goals (in 2015) were launched [8], targeting issues related to poverty, hunger and climate change [9]. In this direction food and nutritional security added another dimension in recent times as holistic development [10].

Present agriculture should be encompassed of all these optimistic targets to nourish the present and to fulfil the requirement of future generations. The GRTs and industrialized agriculture introduced huge use of chemicals, and within a few decades, antagonistic impacts on the environment became prominent. The nitrous oxide (NO_2_), a greenhouse gas, is generated from applied chemical N fertilizers from the soil leading to the deterioration of soil quality and global warming. Additionally, ammonium nitrate (NH_4_NO_3_) released from chemical fertilizers negatively influences soil-microbes and thus reduces symbiosis between plant and rhizospheric microbes. Excess ammonium (NH_4_) present in soil is converted into nitrate (NO_3_) by N-fixing microorganisms [11], which is released into the atmosphere by N_2_O by the process of denitrification, and the liberated N_2_O causes groundwater pollution through the process of eutrophication [12]. Further, the chemical N fertilizer production process releases GHGs in the atmosphere, and to satisfy the current need of global chemical N fertilizers, 300 Tg of CO_2_ per annum is released into the air [13,14]. The contribution of the agriculture sector is in the leading position to use chemical pollutants in the form of chemical fertilizers and plant protection chemicals [15] causing the disturbance in the agroecosystem.

The current agriculture is facing various abnormalities because of global warming and its resultant as climate change. As the climatic parameters as well as abiotic factors very much influence potential yield under a standard package of practices of crops in an agroclimatic zone, climatic abnormalities are of important concern to obtain satisfactory crop productivity. Industrialized agriculture worldwide initiated the use of chemical inputs, improved crop ideotypes and maximum exploitation of natural resources under supply-driven productivity enhancement mode. These fossil-fuel-based high energy inputs ultimately have lost the ecological balance along with the facilitation of pollution. The anthropogenic initiatives in agriculture are also responsible for global warming and climate change. Agriculture alone shares about 47 and 58% of total anthropogenic emissions of CH_4_ and N_2_O, respectively [16]. Similarly, the production of plant protection chemicals, tillage and harvest operations and non-judicious use of chemicals are causing havoc in global warming and climate change. Under the consequences, it is the right time to give an insight into the possibilities of bio-inputs as well as bioinoculants that can perform multifaceted roles by overcoming the issues of contemporary agriculture.

In the populous and developing countries, there is enough need for the production of food and for production with chemical inputs causing the change in soil pH, negative impact on a natural population of soil microbes and degradation of agroecosystem as a whole [8]. For sustainable agricultural production, care should be taken for maintaining soil health [17] by the adoption of eco-friendly technologies [18] including the use of helpful microbes in agriculture [19]. The importance of microbes is well recognized for enhancement of soil health [20] recovery of plant stress [21] and increase in productivity of crops [22,23]. Crops produced should be free from stress, and those of superior in nutritional quality are the prime target to achieve agricultural sustainability and food security. The desired crop qualities are accomplished by exploiting the potential of microorganisms [24,25]. Among different microorganisms, the plant growth-promoting rhizobacteria (PGPR) are prominent and in extensive practice [26,27]. However, microbes have the potential to minimize the abiotic [28,29] and biotic stress [30,31,32] of the plants.

Microbes are delicate organisms and susceptible to environmental changes. Excess use of chemicals to supply nutrients or plant protection can hinder soil biological process and thus may cause a threat to microorganisms [33]. To reduce the harm caused by synthetic and chemicals, alternative inputs of organic and microbial origin can be used which will ensure environmental safety and agricultural sustainability. Many developed countries have already reduced the usage of chemical fertilizers in farming and promoted bio-inputs [34]. Presently, biofertilizers and biopesticides are in use, but on a very low scale. Broadly, these are known as bioinoculants, comprised of living or latent cells of microorganisms that multiply under favourable environments and act positively on plant’s health [35]. The use of bioinoculant in agriculture, not a new intervention, rather a century-old technology and rhizobium inoculation was first described and patented by Nobbe and Hiltner [36]. Basically, the soil is a biological laboratory and harbours various microorganisms [36]. In other words, the bioinoculants are regarded as plant growth-promoting microorganisms (PGPM), because they facilitate plant growth by making essential nutrients available to crops [37,38,39] or providing resistance against different abiotic and biotic factors [40,41,42]. Different inoculants are used individually or in the combination of different types of microorganisms as consortia [43,44,45,46] for evergreen agriculture. Microbial inoculants, because of their multiple roles such as nutrient supplementation, plant growth promotion, soil health enhancement, disease suppression, etc., can provide resilience, especially under harsh environmental conditions. Moreover, unlike fertilizers, biofertilizers do not have any harmful effects on the ecosystem. In this article, the available literature has been carefully synthesized to understand the potential roles of microbial inoculants as next-generation inputs to enhance agricultural sustainability under a changing climatic scenario.

## 2. Green Agriculture, a Paradigm Shift

There are various contemporary and competing thoughts and claims for achieving agricultural sustainability with diversity, resource improvement, resilience and also efficiently productivity. These are resource conservation technology (RCT), for the growth of more productive genetically modified and transgenic crops, organic agriculture, sustainable intensification of agriculture and creation of healthy agro-ecosystem are some promising approaches in this regard [47]. Interestingly, it is true that this low-energy, as well as low-input or market-driven technologies, are unable to fulfil the issues and concerns of marginal and resource-poor agricultural lands of the developed countries and assure agricultural sustainability in a true sense. However, it is obvious that the agricultural production system greatly depends on ecosystem services leading to sustainability. The nourishment of biodiversity is the key to ensure better ecosystem services [48]. Rich biodiversity not only favours the agricultural production system, but also makes many ecosystem services like balance in microbiome population dynamics, micro-climate regulation, influence on the local hydrological cycle, detoxification of poisonous chemicals, etc. In this way, biodiversity maintains biological integrity and a healthy ecosystem, and traditional farming exhibits richness in biodiversity [49,50]. The latest concept indicates a priority on maintenance of local natural resources, which can assure healthy ecological services [51] towards green agriculture. Green agriculture is farming practices that uphold and enhance farm productivity and profitability on a sustainable basis, decrease adverse externalities and reconstruct lost ecological resources with greater efficiency by minimizing pollution [52]. In general, green agriculture is locally adaptable agricultural procedures and authentic market-driven certifications such as Good Agricultural Practices (GAP), organic farming and conservation agriculture and related techniques in which bioinoculants play a very crucial role by maintaining soil health. A change of mind-set and paradigm shift towards green agriculture and green economy may save the agroecosystem from further degradation [53].

## 3. Role of Bioinoculants in Green Agriculture

Bioinoculants are formulations comprised of microbes used as a tool in green agriculture. In phyto-microbiomes, microbes are abundant and they form holobiont in association with plant [54,55,56] and plants flourish with microorganisms association [57,58,59]. The microbes of rhizo-microbiomes play a significant role in the growth and development of plants. Microbes have the capacity to assimilate and acquire essential nutrients of plants, improve soil physicochemical properties, and modulate secondary metabolites, antibiotics, plant hormones and various signal compounds. Additionally, microbes secrete different biostimulants, which play important roles in influencing physiological and metabolic activities [43,60,61,62,63]. Different beneficial microorganisms have been considered for green agriculture to improve nutrient availability in the rhizosphere and uptake, tolerance of abiotic and biotic stress. The distinct roles of microorganisms as bioinoculants in plant health and growth promotion have been discussed in the following sub-headings:

### 3.1. Nutrient Assimilation and Biofortification

Plants require inorganic minerals for their nutrition, growth and development, of which phosphorous (P), nitrogen (N), potassium (K), calcium (Ca), magnesium (Mg) and sulfur (S) are known as macronutrients, while micronutrients are copper (Cu), manganese (Mn), zinc (Zn), boron (B), molybdenum (Mo), iron (Fe) and chlorine (Cl) [64,65]. The micronutrients are found in plant tissues in a very less concentration (<0.01% of the dry weight of plant tissues). Some plants like legumes, wheat, barley, citrus, peach also require nickel (Ni) and the urease enzyme consists of Ni that takes part in the hydrolysis of urea in tissues [66,67,68,69]. There are some more minerals in the list which are found as important to some plants grown in a specific environment and these are aluminium (Al), cobalt (Co), selenium (Se), silicon (Si) and sodium (Na), but the essentiality of these elements has not been recognized [70]. These nutrients are present in nature in different forms and microbial inoculants assimilate and ensure bioavailability to plants [71]. Microorganisms get involved in composite processes in nutrient assimilation in plants and facilitating the growth of the plants [72]. Bioinoculants flourishing in the neighbourhood of roots enhances plant health by increasing uptake and availability of nutrients through the process of N fixation, P solubilization and mobilization, K mobilization and other micronutrient mobilization [73]. Different bioinoculants already tested in earlier studies showed promising results (Table 1).

N is required in the desired quantity to plants because it is constituent of amino acids (used in forming protoplasm), enzymes, chlorophyll and several vitamins and is indispensable for physiological and developmental mechanisms of plants. In absence of enough N in unfertile soil, plants are unable to perform all necessary processes [88]. To fulfil the requirement of N exogenous application of manures and fertilizers are common in crop production. However, N is abundant in the atmosphere and capturing of atmospheric N biologically is possible, in which different microorganisms play a pivotal role and the process is known as biological nitrogen fixation (BNF).

Some bacteria and methanogenic archaea capture N from the atmosphere and offer it to plant roots in a utilizable form [89,90,91]. Different estimates revealed that BNF accounts for about 200 million tonnes annually [92], and it can be a substitute of chemical N inputs if properly managed. The roots of leguminous crops form a nodule in which N fixing bacteria harbour and fix N in symbiotic association and nitrogenase plays a great role in the process [93]. In the symbiotic N fixation process, molecular communication takes place between bacteria and plants in which signals reach to plants through flavonoid and isoflavonoid pathways enticing the rhizospheric bacteria. *Bradyrhizobium*, *Mesorhizobium*, *Rhizobium* and *Sinorhizobium* are common endosymbiont bacteria that respond to the signals and form nodules in the roots of leguminous plants [94]. Both the plants and Rhizobia experience significant transformations as the legume forms nodules and *Rhizobia* altered into a branched bacteroid [95]. *Rhizobium* can fix as much as 100 kg N ha^−1^ annually in a symbiotic association with legumes [96] and Rhizobial N fixation has already been quantified, ranging from 20 to 22 Tg N year^−1^ [97] up to 40 Tg N year^−1^ [12]. *Rhizobium*-legume association is selective and specific *Rhizobium* is associated with distinct legume species [98]. Association of *Rhizobium* with nonlegume plants like *Parasponia* is also noted [99]. Symbiotic association of microorganisms with non-legume plants also occur, and *Frankia* associate with non-leguminous plants. Sometimes, plant *Frankia*-mycorrhiza, a tripartite association, is also noted [100]. Nostoc is cyanobionts, and is known to fix N biologically by the process of heterocysts, akinetes and hormogonia [101]. Other than rhizobia, most of the microorganisms fix comparatively less quantity of atmospheric N by the adoption of different mechanisms [102,103].

Phosphate (P) is another important primary nutrient and deficiency is very common in the soil. P is an essential component of various organic compounds and metabolites like deoxyribonucleic acid (DNA), ribonucleic acid (RNA), phospholipids, sugar phosphates, phosphoproteins, adenosine triphosphate (ATP) and some amino acids. In leguminous plants, P facilitates the increase of nodulation [104,105], synthesis of different acids, protein and oil [106] and P helps glucosinolate formation which enhances oil content in oilseed crops [107,108]. P is absorbed by the plants as H_2_PO_4_^−^ and HPO_4_^=^ and both the orthophosphates are highly soil pH sensitive. Uptake of P is also facilitated by rhizospheric microorganisms, and for green agriculture, in the future, these phosphates solubilizing and mobilizing microorganisms can perform a great role in P nutrition to plants. There are microorganisms abundant in soil that can solubilize and mobilize P remaining in soil in unavailable form and thus plants get nutrition. Some P solubilizing Microorganisms are species of *Agrobacterium*, *Aspergillus*, *Bacillus*, *Burkholderia*, *Enterobacter*, *Erwinia*, *Flavobacterium*, *Micrococcus*, *Pantoea*, *Pseudomonas*, *Rhizobium*, *Penicillium* [109,110,111,112,113]. Among these different strains of *Bacillus* like *B. circulans*, *B. megaterium*, *B. polymyxa*, *B. sircalmous* and *B. subtilis* are important. *Pseudomonas* and *Enterobacter* are also equally important in solubilizing soil P [114,115]. *Rhizobium* isolates from *Cassia absus* and *Vigna trilobata*, *Rhizobium* sp. strain 17, 19 and 26 from *Sesbania sesban* showed solubilization of P [116]. Rhizobial isolates collected from cluster bean (*Cyamopsis tetragonoloba* L.) were noted to solubilize P present in the soil. The existence and volume of PSB in soil depend on soil physicochemical properties, crops raised and cultural practices adopted; however, more PSB population is observed in pasture and agricultural lands [117]. Microbes transform insoluble for of P to orthophosphate, the available form to plants through the processes of synthesis of protons, organic acids, hydroxyl ions, carbon dioxide and siderophores [38,118]. Further, H^+^ translocation of ATPase, direct exchange of H^+^ and phytases and phosphatases based mobilization of P are also noted in soil by microorganisms [45,119,120]. *Arbuscular mycorrhizal* fungi (AMF) can make greater availability of P to plants [121,122]. Other than mobilizing soil P to plants AFM provides tolerance to plants and prevents down-regulation of metabolic pathways [121,123]. AFM are also known to interact with PSB and make P available [122,124]. Direct solubilization of P by Gram-negative bacteria is also observed [125]. Inoculation of all these microorganisms can fulfil the P requirement of plants to a large extent and play a pivotal role in green agriculture.

Another important macronutrient is potassium (K) useful for the life process of the plants. K takes in numerous physiological and metabolic processes such as enzyme activation, seed germination and emergence, stomatal activity, photosynthesis, water and nutrient transport, protein and starch synthesis and quality of crop [126]. The upregulation of potassium reduces reactive oxygen species (ROS) generation and nicotinamide adenine dinucleotide phosphate (NADPH) oxidases [127,128]. K holds photosynthetic electron transport activity, facilitates stimulation of the adenosine triphosphate (ATP) synthase enzyme, influences ATPase activity, helps in tolerance of different abiotic and biotic stresses [129]. Beneficial soil microorganisms like actinomycetes, bacteria and fungi, and actinomycetes take part in the chelation of soil K and facilitate its availability to plants. Different mechanisms involved in the process making K available to plants are chelations, acidolysis, synthesis of polysaccharides, complex and exchange reactions [39,130]. Some important K solubilizing bacteria (KSB) are *Acidothiobacillus ferrooxidans*, *Bacillus circulans*, *B. edaphicus*, *B. mucilaginosus* and *Paenibacillus* spp. [38]. *Frateuria aurantia* is another important KSB [131]. KSB dissolve silicate and release Kand these can be effective bioinoculants for the future. *Aspergillus niger*, *Aspergillus terreus* and *Aspergillus fumigatus* are also having the capacity to solubilize K [132,133]. *Serendipita*, a fungal endophyte, is known to increase the K+ concentration in maize under stress due to salinity [134].

Micronutrient deficiency is a common problem of lands where intensive agriculture is practiced and known as ‘hidden hunger’. The crops produced with deficient nutrients suffer from the nutritional quality and artificially biofortification is done. However, there are microorganisms which can make the bioavailability of micronutrients. Thus desired concentration of nutrients can be obtained in agricultural produces [135]. Iron (Fe) is a micronutrient available in soil as ferrous and ferric forms and plants uptake ferrous ions [136]. Fe acts as a cofactor for cellular respiration, thylakoid biogenesis, oxygen transport, chloroplast development and chlorophyll biosynthesis [137]. In calcareous and well-aerated soils Fe deficiency is a common problem causing chlorosis to plants resulting in loss of crop productivity and quality [137]. Some microorganisms are capable to make availability of ferrous ions in the rhizosphere, and thus the role of these microbes is important in green agriculture [138]. Some low molecular weight organic chelators involved in the chelation of Fe are known as siderophores. In calcareous and well-aerated soils microbes synthesize and release siderophores that make Fe hydroxides soluble [139]. Pseudomonas sp. secreted siderophore has the quality to make Fe available [140]. In legumes, nodulation and symbiosis with Rhizobium can boost Fe nutrition to plants, and [141,142] reported the *Bacillus subtilis* GB03 iron acquirement in *Arabidopsis*.

Zinc (Zn) is another micronutrient needed in less quantity (below 0.2mg g^−1^ dry matter) [143] that performs in various physiological [144] and metabolic processes [145]. Zn helps in water uptake and transport [146] and reduction of stresses like heat [147] and salinity [148]. Zn registered a vital role in the production of auxin hormone, enzymes like aldolases, dehydrogenases, isomerases, RNA and DNA polymerases and transphosphorylases [145,149]. Further, Zn performs protein synthesis [150], lipid metabolisms and nucleic acid synthesis and formation of DNA and RNA [145,151]. Acidic pH makes the availability of Zn, but the presence of more calcium carbonate [152] and phosphate [153] lowers the abundance of Zn. Zn deficiency is a very common problem of the soils where intensive agriculture is practiced and to overcome chemical forms of Zn is applied to the soil which may further create toxicity with the excessive application. On the other hand, there are microorganisms which can make Zn available to plants. Considering the importance of these microbes for future green agriculture proper attention should be given. Soil microbes solubilize Zn by acidification which lowers pH and makes it available. Further protons, siderophores and oxidoreductase reactions on cell membranes also make Zn available to plants [154]. *Pseudomonas fragi, Pantoea dispersa, Pantoea agglomerans,* *Sedum alfredii* are microorganisms known as Zn solubilizers in soil [155,156]. Further, scientists noted the beneficial role of microbes like *Azospirillum* [156], *Bacillus aryabhattai* [157], *Bacillus* sp. [158], *Pseudomonas*, *Rhizobium* strains [159,160], *Pseudomonas aeruginosa* [161], *Serratia liquefaciens*, *S. marcescens*, *Bacillus thuringiensis* [162], *Gluconacetobacter diazotrophicus, Microbacterium saperdae, Enterobacter cancerogens* [163,164], *Bacillus* sp., *Burkholderia cenocepacia*, *Pseudomonas striata*, *Pseudomonas fluorescence*, [165], *Acinetobacter* sp. and *Serratia* sp. [166] in solubilization of Zn.

Manganese (Mn) is another micronutrient for plant growth which performs many roles in physiological and metabolic processes. It is an essential constituent in the structure of photosynthetic proteins and enzymes [167]. Mn also takes part in respiration, pathogen defence, and hormone signalling and foraging of ROS. Mn is available in soil in two forms, Mn^4+^ (oxidized form) and Mn^2+^ (reduced) forms, of which reduced form is used by plants. Microorganisms like AMF can make Mn obtainable to plants [168]. Low temperatures with higher O_2_ concentrations enhance Mn^2+^ oxidising (aerobic) organisms. However, there is further research needed on the identification of suitable Mn^2+^ oxidizing microbial strains and their uses as bioinoculants in future agriculture.

### 3.2. Management of Pests and Pathogens

Like beneficial microorganisms, phytopathogens are also present in the soil and harm to crops. An estimate mentioned that up to 16% yield loss may be caused by disease infection [169,170]. However, FAO [2] estimated 20–40% yield loss at the global scale caused by pests and diseases. To manage pests and diseases, chemical pesticides are generally applied and in developing countries pesticides are used indiscriminately causing harm to the agroecosystem [171]. However, the chemicals applied for plant protection do not only control the pathogen, but also harm to all life forms including non-target and beneficial soil microbiome [172]. A major portion of applied herbicides and insecticides (95 and 98%, respectively) harm to non-target soil microbes [171,173]. Further, chemical pesticides pollute the agroecosystem [174,175] hampering efficient and sustainable ecosystem services [176,177,178,179]. Considering the concept of green ecology as well as green agriculture, weapons for biological control should be the top priority that is nothing but the management of pests population dynamics below the threshold level by using bio-agents [180,181,182]. In this regard, microbes can play a great role. The strategies for bio-control include competition for nutrients, niche exclusion and allelochemicals synthesis [183]. Antibiosis is an important mechanism of the bio-control of pests [184]. Different broad-spectrum antibiotics are produced by microbes, mainly actinobacteria (Table 2).

Microbes produce different enzymes like cellulose, chitinase, glucanase, protease or proteinase and laminarinase, which are involved cell wall hydrolyzing, and these hamper the biological process of the fungal pathogen [207]. Some toxic metabolites (like HCN, Cry protein, exopolysaccharides, biosurfactants, taurosporine, etc.) are also produced by microbes that suppress harmful insect-pest population [208,209,210,211] (and these compounds have insecticidal and larvicidal properties which deserve wide use in green agriculture (Table 3)).

Niche exclusion in the rhizosphere is a common approach of beneficial microbes and thus pathogen population in soil is controlled [212]. These mechanisms are known as beneficial microbe-induced systemic resistance and pathogen-induced systemic acquired resistance [213,214]. Following Table 4 shows a list of the antagonistic microbes and suppressed pathogens (Table 4).

**Table 3:** Antibiotic producing actinobacteria.

Actinobacterial Species	Antibiotic	References
*Streptomyces* sp., *S. alboniger*, *S. padanus*	Alnumycin, coronamycins, fungichromin, goadsporin, kakadumycins, pamamycin- 607, rhodomycin	[215,216,217]
*Micromonosporacarbonacea*	Everninomicin	[218]
*Actinoplanesianthinogenes N.* sp.	Purpuromycin	[219]
*Micromonospora inyoensis*	Sisomicin	[220]
*Actinoplanes*	Lipiarmycin	[221]
*Actinomadura*sp.	Cationomycin, chandrananimycins, oxanthromicin	[222,223]
*Actinoplanesteichomyceticus*	Teichomycins, teicoplanin	[224,225]
*Micromonospora echinospora* sub-sp. *Armeniaca* sub-sp. nov.	Clostomicins	[226]
*A. utahensis*	Echinocandin	[227]
*Actinomaduras piralis*	Pyralomicins	[228]
*Microbispora* sp.	Cochinmicins, glucosylquestiomycin	[229,230]
*Micromonospora lomaivitiensis*	Lomaiviticins A and B	[231]
*Actinoplanesfriuliensis* sp. nov. *II.*	Friulimicins	[232]
*Microbisporaaerata*	Microbiaeratin	[233]
*Nocardia* sp. *I*.	Nocathiacins	[234]
*Nocardiamediterranei* subsp. *kanglensis*	Chemomicin A	[235]
*Nocardiopsis*	New thiopeptide antibiotic	[236]

### 3.3. Abiotic Stress Management

Plants suffer due to different abiotic stresses. Among different abiotic stresses, the problem of soil salinity is very crucial. Soil salinity is characterized by a high concentration of neutral soluble salts, such as chloride and sulphates of sodium. When the electrical conductivity (EC) of the soil is >4 dS/m that creates an osmotic pressure of 0.2 MPa is called saline soil [245]. Salinity stress that brings about the osmotic stress inhibits or slows down the water and nutrient absorption through plant roots resulting in stress [246]. Higher absorption of sodium and chlorine is observed under salinity conditions. However; a deficiency of calcium and potassium is observed. Such deficient or excessive absorption of nutrients leads to a nutrient imbalance in plants [247]; leading to specific ion toxicity and osmotic stress. Salinity stress also leads to oxidative stress due to the production of reactive oxygen species (ROS) [248], which causes cellular damage through membrane disintegration, which ultimately harms crops [249,250]. The salinity affected plants show distinct symptoms such as scorching in leaf blades, mottling of leaf and necrosis. Salinity brings down photosynthesis, and growth is also reduced [251,252]. Some volatile organic compounds (VOCs) such as 2-pentylfuran, 3-hydroxy-2-butanone (acetoin), dimethyl hexadecyl mine and 2,3-butanediol released by microorganisms, namely, *Alternaria*, *Arthrobacter*, *Bacillus*, *Pseudomonas and Fusarium,* promoted growth under different abiotic stress conditions [253]. Microorganisms like *Bacillus subtilis* GB03 enhanced tolerance against salinity to rockcress (*Arabidopsis thaliana*) [254,255], however, Vaishnav et al. [256] noted increased tolerance to salinity by soybean (*Glycine max*) due to the presence of bacterial strain *Pseudomonas simiae*-4. Singh [257] reported that *Cyanobacteria* favoured the growth of field crops, namely, rice, wheat, maize and cotton. A bacterial strain (*Pseudomonas koreensis* strain AK-1) helped in the reduction of NaCl level in soil and thus, facilitated the growth of soybean [258].

The impact of drought stress might vary depending on the duration of exposure to the stress, intensity or severity of the stress, and the time period of the plant’s life cycle during which the plant is exposed to the stress [259]. Drought causes about 30–50% yield reduction owing to high evapotranspiration loss and low water availability, increased respiration and enzyme activity in the plants [260,261]. Drought also leads to a reduction in carbon assimilation via photosynthesis (which might be due to partial or full closure of stomata in response to water stress, which also reduces the entry of carbon dioxide into the leaf, lower activity of enzymes involved in photosynthesis due to water stress, etc.) [262], water loss and phosphorus and nitrogen content in tissues of plants [263,264]. A reduction in growth characters was also observed by [265,266]. Under drought stress, starch is converted to sugar [267,268]. Further, amino acids and polyamine synthesis were also affected by the drought [269]. Figueiredo et al. [270] demonstrated that *Rhizobium tropici* and *Paenibacillus polymyxa* were involved in the upregulation of genes responsible for stress tolerance in kidney beans (*Phaseolus vulgaris*). Wang et al. [271] noted that production of proline, monodehydro ascorbate, and gene expression in cucumber (*Cucumis sativa*) by *B. Serratia* sp. XY21, *B. subtilis* SM21 and *Bacillus cereus* AR156, and which reduced drought stress. AMF inoculation in soybean increased proline concentration in soybean that reduced stress due to drought [272].

Like any other stress, the effect of heat stress varies with the intensity and duration of the stress. Temperature above 30 °C usually results in water deficiency and may limit plant growth, especially when the soil is dry, humidity is low and the evapotranspiration demand is not met [273,274,275]. However, the response to the heat stress may vary from crop to crop. In maize, photosynthesis is inhibited, when temperature rises above 38 °C. The impact is more when temperature increase was suddenly rather than gradually [276,277]. Considering the fact that both heat and drought stress negatively affects the water balance of plants, hence both the stress negatively affect nutrient uptake and photosynthesis. Spikelet sterility in rice is observed due to high temperatures that lead to the production of chaffy grains, which ultimately reduces the crop yield [278]. Drought and heat stress together causes quick water loss from soil (evaporation loss) and plant surfaces (transpiration) [279]. Insufficient water supply to fulfil the evaporative demand can lead to heat stress [280,281]. De Zelicourt et al. [282] observed that colonization *Curvularia proturberata* isolate Cp4666D in roots enhanced heat tolerance in Woolly rosette grass and tomato. *Bacillus amyloliquefaciens* and *Azospirillum brasilence* assured reduced regeneration of ROS, pre-activation of heat shock transcription factors and changes in metabolome in wheat.

Heavy metals are higher density elements and cause toxicity at a low concentration [283]. The non-judicious fertilizer and agrochemical application, application of a huge amount of sewage and sludge contaminated with heavy metals, smelter dust and industrial are the major causes of heavy metal pollution [284]. Lead and cadmium substitution in biomolecules result in the inhibition of growth due to metabolic disturbances [285]. Adaptation mechanisms for tolerance to heavy metals include protein repairing, metal chelation, cell wall binding, metal pumping, subcellular compartmentation, etc. [284,285,286,287]. Heavy metals may damage cellular structures. Some examples include, DNA damage in leaf and root tip of *Vicia faba* due to metal stress [288], DNA damage by cadmium interfering transcription [289], damage in a photosynthetic protein complex and decreased Hill reaction on increasing Ni concentration in *Zea mays* [290], injury to macro-molecules on the formation of ROS [291,292], chloroplast structure alteration and activity of PS II affected [293] due to dissociation of O_2_-evolving complex, reduction in chlorophyll molecule with more concentration of Zn [294], unstructured changes in chloroplast due to Cr toxicity, decreased water use efficiency (WUE) and rate of photosynthesis due to Pb toxicity [295]. Plants grown in heavy metal contaminated soil shows poor growth owing to poor photosynthesis resulting from damage in the photosynthetic apparatus. Disruption in the electron transport chain and production of ROS also damages cellular organelles and cell membranes [296,297]. ROS is considered a “double-sword” in plant physiology [298], as it results in oxidative damage to the membranes/tissues as well as signal important developmental processes [299], cell wall modification [300], transcriptional activities [301] and protein kinase cascade [302]. Plants produce ROS in response to heavy metal stress [303]. To counteract the negative impact of excessive ROS production and to maintain cellular homeostasis, enzymatic antioxidants such as SOD, CAT, APX, and GR are activated by plants. Many non-enzymatic antioxidants such as ascorbic acid, proline, phenolics, α- tocopherol, carotenoids, AsA, GSH, flavonoids, etc., also play a major role [304,305,306]. Arsenic toxicity was reduced in Indian mustard (*Brassica juncea*) by *Staphylococcus arlettae* due to induction of increase in soil dehydrogenase, available phosphorus and phosphatase [307]. Ma et al. [308] demonstrated resistance to Pb, Zn and Cd toxicity up to an elevated extent due to inoculation of *Phyllobacterium myrsinacearum*. In another study, *Pseudomonas brassicacearum* and *Rhizobium leguminosarum* increased metal-chelating molecules in Indian mustard [309].

As heavy metal contamination is a growing problem across the globe, hence finding suitable strains of microorganisms capable of detoxification or decontamination of the heavy metals can help in improving crop productivity. Isolation and culture of the microbes capable of remediating heavy metal contaminated soil is one of the most promising areas in microbiological science which needs to be explored further.

## 4. Microbe Based Inoculant

In the late 1960s, agricultural research focussed on crop efficiency, high-yielding varieties, irrigation systems, agrochemicals, etc. About half of the yield development was being done by the utilization of fertilizers alone. Agricultural practices are subjected to the utilization of pesticides, inorganic fertilizers and other agrochemicals, which have raised the farm production; however, they have brought about the exhaustion of natural assets, ecological crumbling, and environmental pollution. Inorganic fertilizers are considered as one of the significant operators for causing soil contamination. A requirement for sustainable farming can be accomplished by building the rhizospheric microflora. Sustainable agriculture is of enormous importance in the present context of continuously dwindling natural resources and it offers the possibility to meet the future agricultural needs which are not given by industrialised agriculture. Sustainable agriculture is the amalgamation of the following destinations:

○ Financial gain,
○ Natural wellbeing,
○ Social and monetary value,
○ Protection of condition,
○ Productive utilization of non-renewable assets.

Sustainable farming approaches aim to build harmony among soil, crops and their associated environment. Soil is considered to be the most fragile living mediums, which must be protected to ensure profitability and productivity. Soil has been found to be a unique biological system that acts as a medium to vegetation. It comprises natural matters, minerals, and different life forms. Various microorganisms stay richly in the soil. Microorganisms occupying the rhizosphere area of soil assume a cardinal job in agriculture by advancing the trading of plant nutrients and diminishing the utilization of concoction composts to an enormous degree. There are a few activities by which microorganisms that are rhizospheric improve plant development [310]. Microflora from soil, for the most part, comprises of free-living microorganisms, for example, parasites, actinomycetes, PGPR, PSB, and AM growths. Every one of these living beings adds to the development of different plants and improve soil fertility. The natural exudates discharged from the plant acts as nutrients to the microbial network present in the rhizospheric soil. The root exudates fill the double need of expanding microbial populace alongside improving the soil structure.

To counter the negative effect of excessive agrochemical use, the sustainable agriculture approach aims at responsible use of all available resources to maintain and enhance agroecosystem health. To decrease the use of synthetic compounds, the expectation for higher profitability, the PGPB have been found very promising [311,312,313]. Microorganisms residing in the rhizospheric zone or at their site of action brings about multiple beneficial effects that help in the improvement of soil and plant health [314]. A few microorganisms may benefit from root exudates [315]. Similarly, endophytic microorganisms may get a favourable environment, by colonizing within plant tissues, because of natural association. Even though there are the significance of types of microbes and the site of colonization, choosing the correct microbes is basic, with qualities of interest, as indicated by the objective, regardless of whether, e.g., they are biocontrol agents, biofertilizer or phyto-stimulators [316].

The microorganisms occupying the rhizosphere are involved in imparting the resistance in a plant and also involved in nutrient absorption and uptake [317,318]. Understanding the role of individual microorganisms and their activity can help in planning the application of microbial consortia in a way that can help in improving soil health and improving crop productivity. The improvement in crop growth might be due to plant growth-promoting activity, improving nutrient absorption, solubilization and mobilization, suppression of harmful or pathogenic microorganisms, etc. [319,320]. These are the few components by which microorganisms invigorate the development of plants. A few logical articles support the confirmed impacts of microbial immunization on the advancement of the development of plants.

Biofertilizers are found to be a class that improves agricultural productivity and/or soil health with their bioactivity [321]. Biofertilizers and biopesticides hold the possibility to improve the agriculture production system with a suitable methodology [322]. These are being connected normally to the development of the plant, advancement and reactions to abiotic stresses, actuated by mixed bioactive materials from an incredible assorted variety. The helpful microscopic organisms can produce phytohormones and different mixes [323], biomasses and their concentrates, e.g., green growth [324] and yeast [325], or by mycorrhizal growths [326], even items acquired [327], among an immense decent variety of sources from biotechnology and nature. In a similar idea, the biopesticides characterized by the US Environmental Protection Agency (EPA) as pesticides produced from characteristic materials [323], are non-pathogenic strains of microorganism [328] or extricates of plants [329], impact against pests or ailments, or the bioinoculants identified with biologic nitrogen take-up, are called as biofertilizers as well. Brazilian guideline decides the bioactivity as a principle impact: “Biofertilizer is an item that contains dynamic fixing or natural operator, free for agrochemicals, fit for act legitimately or by implication on all or part of developed plants, raising the efficiency, without considering their hormonal or invigorating worth” [328]. In the guidelines of Brazil’s natural creation, biofertilizer is defined as an “item containing dynamic segments or organic specialists able to acting, legitimately or in a roundabout way, overall or part of developed plants, improving the exhibition of the creative framework and that been liberated from substances precluded by the principles of natural creation” [330]. In the two guidelines, the bioactivity, as well as some dynamic fixings, is expected to portray a biofertilizer.

**Figure 2:**
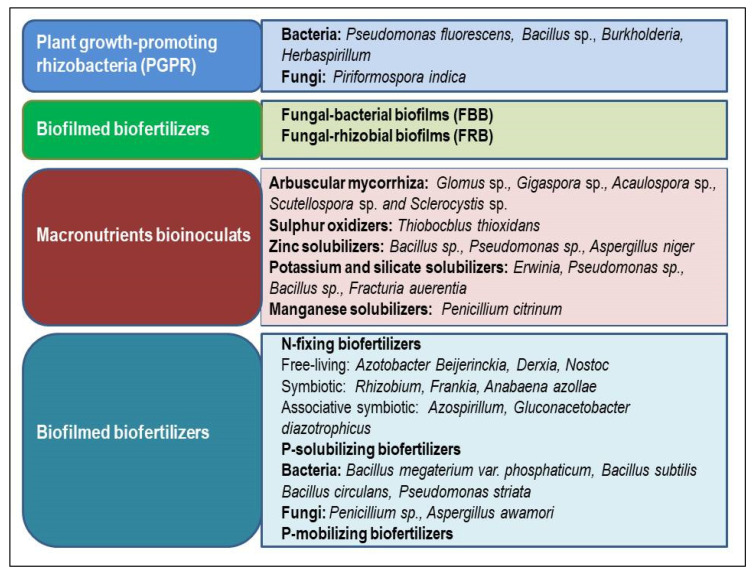
Prospective bioinoculants which may be used in green agriculture.

Biofertilizers contain different living organisms that, when given to surfaces of the plants, seed, and soil, quicken their procedures that bring about the accessibility of nutrients for simple absorption [331,332,333,334,335] (Figure 2). As nitrogen is latent in nature, the plants can’t use it directly. At surrounding of different conditions, diazotrophs intercede obsession of nitrogen under different enzymatic responses by a procedure called as biological nitrogen fixation.

### 4.1. Bacteria

Species of bacteria might be defined as a gathering of comparable different genomic strains, who shared higher comparability in numerous autonomous attributes [336]. The comparability between prokaryotic to be viewed as an animal category must be more prominent than 97% of the 16S ribosomal quality arrangement contrasted with the sort strain, permitting microbiologists to quickly distinguish new species [337,338]. In this manner, different qualities have been proposed to acknowledge phylogenetic investigation, where by and large qualities of advancement rate higher than 16S rRNA were used; however, they were being saved to keep up hereditary data to be classified systematically [339,340]. A couple of instances of those qualities are dna K, rec A, atp A, glt A, rpo An, and gln II [341,342]. All necessities of determination of the successions, the different dissemination in a taxon ought to be thought of as just being available in a solitary genome duplicate [338,343]; to acknowledge phylogenetic investigation, multi-locus grouping examinations have utilized in some way *R. leucaenae, Bacillus, Burkholderia, Mycobacterium, Vibrio, Ensifer, Mycobacterium, Rhizobium tropici, Mesorhizobium,* and *R. freirei* [344,345], diminishing vague prospects brought about by hereditary recombination and explicit determination.

#### 4.1.1. *Azotobactor*

*Azotobacter* has been found to be an important biofertilizer in both nonleguminous and leguminous crops and adds nitrogen to the soil to the tune of 25–35 kg/ha. Attempts have been made for large scale manufacturing of *Azotobacte*r and phosphobacteria utilizing the explicit vehicle of the media for phosphobacteria, i.e., Pikovskaya’s media, and for *Azatobactor*, Ashby’s agar [346]. At first, these living beings were separated from different areas, after the finishing of adaptation using different tests; the tally of cells came to 10^8^–10^9^ cells mL^−1^, after the stock was utilized as inoculants.

#### 4.1.2. *Rhizobium*

The most significant of the different biofertilizers is *Rhizobia*, which has been found to be involved in the fixation of the environmental N_2_ by shaping the knobs of roots that go about as smaller than usual industrial facilities of nitrogen obsession in vegetable plants. Atmospheric nitrogen fixation is completed by the enzyme nitrogenase of *Rhizobium* with the assistance of nodules, a protein, and moves towards plants with a viable beneficial interaction [347]. Furthermore, supplements, i.e., phosphorus, potassium, calcium, magnesium and even iron amassing were observed [334,348,349,350,351,352,353].

#### 4.1.3. *Azospirillum*

*Azospirillum* has been found to be colonized in the zone of the roots, and also fixed the nitrogen-free relationship with C4 plants, for example, sorghum, maize, sugarcane, and so forth [347]. This fixes atmospheric N_2_, mineralizes different soil nutrients, iron deposition, and furthermore, favours plant mycorrhiza. The biofertilizers were found to be suitable for C_4_ yield enhancement.

### 4.2. Fungi

In contrast to different microorganisms, these organisms have an unpredictable existence with two morphotypes, anamorphs (abiogenetic stage) and teleomorphs (sexual stage). With a few strains of the structure of the parasite, it is possible that one or both the phases throughout the life cycle are dependent on the above perceptions, and taxonomists created rules of contagious classification. Subsequently, four significant phyla were known, *Ascomycetes, Basidiomycetes, Oomycetes* and *zygomycetes*. The parasitic strains that did not shape any sexual reproduction were found and were classified under a different phylum, *Deuteromycetes*. With the improvement of atomic procedures, it got simpler to allocate an anamorphic stage growth to its teleomorphic stage utilizing inner translated spacer (ITS) area sequencing. Along these lines, with time, the class *Deuteromycetes* bit by bit became out of date. In addition, numerous parasites beforehand having a place with kingdom growths have been situated in either different kingdoms or other phyla. For instance, *Oomycetes* are currently positioned in the kingdom *Chromista*. True organisms contain the accompanying *phyla, Microsporidia Basidiomycota, Glomeromycota, Ascomycota, Kickxellomycota, Blastocladiomycota, Mucoromycota, Entomophthoromycota*, *Chytridiomycota* and *Neocallimastigomycota*. The occurrence of contagious plant infections is expanding the world over. It has been discovered that environmental changes, vulnerable varieties and destructive parasitic pathogens have been assumed to have a significant job in the spread of contagious infections. There are three central matters identified with the investigation of plant pathology:

○ Side effect-based recognition of contagious pathogens,
○ Location of non-indicative pathogens and inactive or tranquil symptoms causing growths,
○ Confirmation or recognizable proof of parasitic pathogens utilizing fitting apparatuses.

Essentially, before the utilization of parasitic strains as biocontrol operators or in bioinoculant, detailing their bona fide recognizable proof is an absolute necessity. Systematics is the investigation of natural assorted variety which incorporates scientific classification, terminology and phylogeny. Those three head systematic divisions direct the portrayal, classification and classification of an organism. For instance, *Phytophthora* recommends a possible pathogen to a non-pathogenic cellulolytic organism [354]. Any adjustment in the growth must influence the character hence influencing the personality of a plant pathogen or a biomanure operator. In this way, genuine distinguishing proof of parasites is significant for controlling the infections, knowing science of the pathogen, component of spreading of different malady, the understanding right character of pathogens where different organisms gave the same side effects, measuring the estimation of the pathogen of ailment misfortune, surveying different varieties in strains, recognizing pathogens and determination of the better biocontrol operators. Pathogens may be available in different environments like in seeds, plant leaves, and soils, and may move via water and air from invaded regions to unwarmed territory.

Besides, the deficient and wrong database of reference groupings additionally presents issues in the right identification [355]. In the yeasts, a large subunit which is around 1400 bp long, is considered as the essential locale for arrangement examination. In any case, D1 and D2 districts of LSU those were considered as hyper-variable areas were normally utilized. A few different areas have additionally been utilized for complete characterisation and better goals at the level of species. Those areas incorporate atomic and mitochondrial rDNA locales 18S ribosomal RNA little subunit (18S-SSU), and 28S ribosomal RNA huge subunit, inside interpreted spacer, intergenic spacer district, mtSSU, and mtLSU, just as protein-coding qualities, for example, RNA polymerase II, β-tubulin, calmodulin, γ-actin, ATP synthase, ef-1α, and so on. Multilocus arrangement composing has been found to be a convenient device to portray different growths like *Chaetomium, Botryosphaeria* and *Alternaria* [118,356,357]. These MLST sequencings have assisted with isolating a few secretive animal categories in *Aspergillus, Fusarium, Penicillium*, *Trichoderma,* etc., the different species ideas utilized in the characterisation of contagious species. In any case, the database for commit growths is restricted [358]. As indicated by Shivas et al. [359], just rust parasites (310 LSU arrangements and 210 ITS successions) and muck organisms (346 ITS groupings) have reference arrangements in GenBank. The successions are utilized to develop a phylogenetic tree dependent on various calculations (greatest miserliness, most extreme probability or neighbour joining) to ascertain the transformative separation between various parasitic species.

### 4.3. Endophytes

Endophytic colonization happens in a few different ways in plants. This course of colonization is by all accounts the rhizosphere, which the organisms reach by chemotaxis and also join to the tissues of plants either by lipopolysaccharide, pili, or maybe exopolysaccharide in cell divider [360,361]. These endophytes, the colonizer of the rhizosphere, connect to the apical roots and enter through a pathway. Ideally, this happens in epidermal intercellular spaces and to the zone of separation [362]. At the point when microscopic organisms enter into the exodermal obstruction, three spots are identified where they can live, viz., inside the cortex, the site of the section, and at the intercellular space. Just a few enter the endodermal obstruction and attack the vessels of the xylem [363]. The endophytes that were approved in the magnifying lens were known as putative endophytes. The endophytes intercede the plant guard in two different ways:

○ The natural endophytic network that ought to contain obstruction skilled attributes, and
○ Restoring of intrinsic endophytic bacterial subpopulations by an approaching bacterium (e.g., a biocontrol operator) [364].

Endophytes are pulled in consideration of analysts to assess them as a potential, increasingly successful alternative for being used as plant development advancement, natural control operators in agriculture framework. On microorganism colonization in plants, they can affect the development. A few gatherings report the instrument of these to be comparable as rhizobacteria, yet scarcely any systems are being demonstrated in plants. These sections will survey the various normal instruments for biocontrol and PGP.

#### 4.3.1. Bacterial Endophytes

Endophytic microscopic organisms have been accounted for availability to the stems, seeds, roots, leaves, tuber, ovule, natural products, and also in vegetable knobs [315]. This populace of endophytic microbes’ shifts rely upon the microbes, and the host have the stage of formation, and also the thickness of inoculation in ecology [365]. These were ruling in plants were being seriously explored [366]. However, this network synthesis was non-definable yet may be dictated to the process of colony formation. Different factors, for example, nature, host phase, the status of physiology, tissue of plants, conditions of soil also practice of farming decide colonization of the microbes [367]. These endophytes were having specific, as an instance, clostridia gathering can be seen as just in a species of grass, i.e., *Miscanthus sinensis* [368]. Bacterial endophytic organisms were found in vegetable knobs as coinhabitants [369]. These were accounted for confinement from various nonvascular and vascular plants signifying a range of endophytic microorganisms [370]. The approach of metagenomics is the ongoing problem area in endophytes [371,372,373,374].

#### 4.3.2. Fungal Endocytes

The fungal endophytes can work opposite to pathogens [375], the herbivores [376], stresses and substantial metals [377], temperature and saltiness [378]. Contagious endophytes are not at all like mycorrhizal parasites, which form a colony in plant roots to develop into the rhizosphere. The tissues of plants are living arrangements for these parasitic endophytes that can develop in any or all plant pieces. Various studies have recorded this nearness of contagious endophytes in unmistakable phyla. Further, more than one parasitic endophyte can be found [379]. Contagious endophytes are dominatingly seen as present in tropical, subtropical, and earthbound biological systems. Likewise, it announced the detachment of all-out 149 parasitic endophytic disconnects having a place with 17 contagious genera in stem, leaf petiole [380]. In the tissues contemplated, leaves appeared around 70% endomycobiota, contrasted with petiole and stem that were 66% and 25.53%, individually. The prevalent genera incorporate *Cryptosporiopsis lunata* (4.18%), *F. roseum* (4.07%), *A. niger* (5.93%), *Stenella agalis* (5.20%), *Fusarium oxysporum* (5.20%)*,* and *Aspergillus interchange* (6.29%).

### 4.4. Effective Microorganisms

Effective microorganisms (EM) are microbial inoculants that stimulate plant growth and soil fertility in agriculture. Generally, EM suspension contains a group of microorganisms such as lactic acid bacteria, yeast, and photosynthetic bacteria. These microorganisms have unique activity by virtue of which they help in improving plant growth. For example, lactic acid bacteria produce lactic acid and suppress many pathogens. The application of EM has been found to improve soil health and soil biodiversity. It has also been reported to improve plant growth, yield and nutritional quality of crops [381,382]. The application of EMs has also been found for composting [382] and water quality improvement [383].

## 5. Next Generation Bioinoculants

Biosafety mirrors a thought of playing it safe to maintain a strategic distance from immense loss of organic uprightness, particularly for human and environmental prosperity. As such, those can be a measurement of prevention, concealment directors, and improve for proper upliftment and to maintain a strategic distance from unintended release of bioinoculants in nature. In recent days, biosafety has become an undetachable piece from the general public thinking about the improvement of humanity. The prime worry of this recognition is the chance appraisal of LMOs and GMOs got through present-day biotechnology. This Protocol of Cartagena for Biosafety depicts, recently shaped biotechnological items ought to follow the measurement of biosafety taking into account for general wellbeing also the monetary benefits if needed, particularly in creating countries. This “prudent norm” used uniquely against the hurtful life form and various countries have the opportunity to enact the limitations that are useful to make sure about the populace and condition. Singular microorganisms have been classified by every nation based on their pathogenicity, methods of transmission, and host scope of the creature, and this order differed from nation to nation on resistance, thickness/development of host populace, nearness of suitable vectors, and guidelines of ecological cleanliness [384]. Sooner rather than later, a significant spotlight will be on the safe-eco-accommodating methodologies by including valuable microorganisms to the system of farming. The vast majority of these potential bioinoculants are generally separated from common fortunes, for example, soil, water, plant, and so on. In any case, in not many cases, organisms which are found to be beneficial can impact to the undesired host. Not many known potential bioinoculants have a place with the genera *Acinetobacter*, *Enterobacter*, *Stenotrophomonas*, etc. Even though not all individuals from these genera have been found to be pathogenic, a few of these have deft pathogens and can cause disease. Additionally, researchers investigated some newly found microbial species isolated from plants and soil, and raised the populace to a limited level for the accomplishment of an ideal advancing impact on plant development that can prompt these destructive impacts to the human condition wellbeing [384]. From this assent, use of novel bioinoculum is perhaps in need of a standard measurement to satisfy important safeguards. The populace pressure-driven need of higher creation and great nature of food inclines toward ecological agreeable editing frameworks, where administrative structure ought to be suggested taking into account appropriate utilization of novel bioinoculants strains as biofertilizers and biopesticides. Other than advancing plant development, bioinoculants can reduce biotic just as abiotic weights on crops, in this manner, giving an ecological agreeable sound option for feasible horticulture. Be that as it may, effective execution of microbial bioinoculants is subject to a timeframe of realistic usability, variable viability across situations and various species of plants other than the structure of soils. Additionally, irregularity in bioinoculants execution, absence of autonomous approval does a bit to assemble trust in adequacy. In this manner, increasingly basic information is required about microbial conduct and associations alongside elements of edaphic and biotic variables for manageable horticulture. For a long while, it is been directed that microbial inoculants for specific types of soil are the superior methodology.

### Constraint-Based Modelling

Metabolic recreation is a long procedure that includes a few parts of the system of metabolism, viz. the proteins, metabolites, and qualities that partake in the metabolic movement, and they are perceived, arranged and associated with the development of a metabolic system. Reliably, the frameworks of a cell are expanding on the basis of genomic grouping and fuse into many responses that participate in the metabolic action of a cell. The data pretty much all metabolic recreation and its product with those assets that help in metabolic investigation and reproduction is introduced.

It is accounted for that CBM watched all the capacity of metabolic systems on concoction physical imperative. A metabolic reproduction gives an away from of the system geography by which genome-scale models can be inferred and utilized for impersonating another distinctive metabolic system of organisms [385]. Scientific displaying and frameworks science approaches could likewise prompt the advancement of novel systems to control the different illnesses in plants by hereditarily changed living beings (GMOs) [386]. It is additionally revealed that OptStrain helps in the fuse of novel protein-coding qualities from various species to a particular microbial genomic organization. As of late, OptReg requirement-based demonstration was created, for the control of down and up-regulation of metabolic chemicals and for the quality knockouts to satisfy the ideal metabolite creation [387]. By bioinformatics studies, quality comments of protein arrangements can be obtained for a huge number of life forms, by which remaking of a metabolic system should be possible. Alongside CBA, flux-based investigations can likewise be applied for the study of metabolic systems. A few scientists detailed that the investigation of fluxes gives rousing experiences without far-reaching active data [388].

## 6. Future Scope of Bioinoculants in Green Agriculture

In the agro-business, the inoculant which can be a microbe, used as biopesticides and biofertilizers have been broadly acknowledged and utilized everywhere worldwide. Those have become the prime decision and ready to contend their ordinary equipped, i.e., concoction based composts, also the pesticides, because of their condition friendly properties. In spite of the fact that having a moderate method of activity, bioinoculants hold rank in consumer decision, might be because of their value as a regular scrounger, which assists with making condition clean. They likewise can possibly help plants to use the most extreme agrochemicals accessible in the field. In a large portion of the cases, bioinoculants were not being found hurtful to purchasers, just the rhizospheric microbes that should be found advantageous in the growth and development of plants. The plants were normally expected to enlist the particular rhizospheric microbiome to drive for plant health and antagonize the harmful microbial populace. Those are likewise found to help use extra natural wastage through treating the soil, particularly when metropolitan natural waste is changed with horticultural soil to advance natural substance. While managing the bioinoculants, much-needed interest should be for fitting the thickness of inoculums accomplishment and level of performance should also be assessed. Taking into account manageable and long haul utilization of biopesticides and fertilizers in the agriculture industry, the following should be the interest of the accompanying problems:

○ Extensive exploration for screening multifunctional and predictable microbial strains, which can be utilized in various rhizospheres;
○ Unravelling the benefits of plant-microorganism collaborations and endeavours to make it progressively useful;
○ Evaluation of valuable microbial strains for equipotential under biotic and abiotic stresses;
○ Monitoring of inoculant for endurance and dispersal in rewarded soil for guaranteed execution;
○ Regulated and careful utilization of biofertilizers as far as creation quality and application.

### 6.1. Improved Root Colonization Ability

Not many microorganisms have been seen as valuable for the plants not being an advantageous connection can be called as PGPR. This plant development was upregulated with the hindering of the development of pathogenic microorganisms [92]. As of late, genetically improved PGPR was being utilized for these pathogens to be controlled and also in the improvement of capacity to develop in the conditions of the restriction of the nutrients. Those corrections upgrade the capacity of the root of the PGPR [56]. Genetic mutations can change the strains of the bacteria and give those the capacity to corrupt the naphthalene and salicylate. Additionally, the inclusion of defreezing quality into the PGPR has empowered it to endure and multiply in cold and play out this biocontrol action [389].

### 6.2. Improvement in Synthesis of Lytic Enzymes

Mycoparasitism has been found to be an instrument utilized to control phytopathogenic organisms biologically. Protease, glucanase and chitinase have been found as the chemicals of lytic enzymes combined with the bacteria just as contagious operators to corrupt the cell mass of organisms, which were anti-development and can cause death [390]. In previous years, scarcely any broad examination has done to get the clone and encoding quality of the lytic compound. These assembled data from different strategies can be a help to improve the nature of biocontrol agents [391]. Qualities that encode the chitinase chemical were cloned in assorted organisms like *Serratia marcescens, Trichoderma harzianum,* and *Enterobacter agglomerans*. Those strains which were being changed have been found to work opposite the phytopathogenic organisms [392].

### 6.3. Improvement in Antibiotic Synthesis

As of late, qualities encoding for delivering the antimicrobials were recognized that can be successful biocontrols of phytopathogens. This accessibility of the antimicrobial qualities may help to improve the biocontrol specialists [393]. Different resources, wherein cosmid pME3090 was embedded in *Pseudomonas fluorescens*, CHA0 brought about the expansion creation of 2,4-diacetylphloroglucinol and pyoluteorin anti-infection agents [394]. Additionally, the hereditarily adjusted *Pseudomonas fluorescens* BL915 strain demonstrated the expanded creation of pyrrolnitrin anti-infection [395].

### 6.4. Improvement in the Synthesis of the Siderophore

For a long time, siderophore production has broadly been investigated. Siderophores can be defined as iron-chelating mixes. Sub-atomic proof acquired from parasitic quality did not indicate siderophore quality [396]. As of late, researchers found that pseudo action MT3A, a receptor in *Pseudomonas sp*. M114, a siderophore that is not orchestrated by *Pseudomonas sp*. This is a proposal of utilizing heterologous siderophores, which can offer an upper hand quality to rhizospheric organisms [397].

### 6.5. Improvement in Synthesis in Bacteriocins

*Agrobacterium radiobacter*, bacteriocin “agrocin 84” have been found to be constrained for the different diseases in plants and is also found to direct the phytopathogens’ development. These gathering analysts utilized a plasmid named pAgK84, which contains agrocin 84-encoding quality, an insusceptibility quality of bacteriocin, and a complex exchange quality. This exchange of pAgK84 to *Agrobacterium radiobacter* brought about a new improvement in the strain of *A. radiobacter* to agrocin84 [398]. The elective methodology has to improve the effectiveness of bacteriocin-orchestrating biocontrol operators by means of increasing the release of bacteriocin or by developing a biocontrol strain facilitating the release of bacteriocin [399].

### 6.6. Developing Hypovirulence by Hereditary Modification

Hypo-destructive strains were seen as liable for spreading sicknesses in nursery crops and chestnuts [400]. These days, the concentration is the hereditarily built *Cryphonectria parasitica* (Murrill) Barr. An alternate methodology can be the inclusion of manufactured transcripts causing infection or acceptance of changes in mitochondria or atomic DNA [401,402].

### 6.7. Resistance Development against Fungicides by Hereditary Modification

At the point where plants have been found to be exceptionally inclined in sickness, this parasitic organic biocontrol gets incapable. Therefore, there is a requirement of the control of disease by different methodologies [403]. Thus, changing this hereditary material, consolidation of fungicide resilience quality of biocontrol specialists improves its action [404]. One investigation where beams of UV have been utilized in the form of benomyl-safe in Fusarium detach, which were working opposite in Fusarium shrink [405].

As agriculture has been found to be the most beneficial practice for humankind, it relies significantly upon the condition of climate and soil. The microorganisms which are found to be based on the plant root region are commonly called the rhizobacteria. The found microorganism has different uses, for example, nitrogen fixation, phosphate solubilisation, production of anti-microbial substances, secondary metabolite, plant growth regulators, auxins, siderophores, etc., which are viewed as critical for plant development. Microorganisms go about as normal foragers because of their capacity to debase dead plant and creature matter, poisons like pesticides, hydrocarbons, colours, paints, and so forth. These organisms can perform all the more viably when included with the wanted sort of microorganism in its dynamic structure and proper amount [406]. This similarity between the strains additionally influences execution, when organisms were presented constraints of having different strains. In prior investigations, these have confirmed, organisms can trigger plant resistant frameworks, viz., incited foundational obstruction (ISR) and fundamental procured opposition (SAR) particularly within plant pathogens sights. These organisms help to stifle the pathogens, in a roundabout way advance plant development. The soil-pull interface gives layers to the intuitive relationship of soil organisms, roots of the plants. This heterotrophic populace of microbes uses exudates of root and decomposes to a carbon source. The rhizosphere and rhizoplane are viewed as encircled with a higher microbial populace contrasted with the soil having no vegetation because of raised degrees of enticing substances, for example, sugars, natural acids, amino acids, nutrients, and so on emitted by plant roots. These substances incite rivalry and pull in organisms of different species, which prompt an assorted microbial populace at various rhizospheres.

## 7. Conclusions

Microorganisms, because of their vast diversity, the multiplicity of roles, wider range of ecological amplitude and host preference, require thorough examination before suggesting their use as bioinoculants. Location and crop-specific studies will help in understanding their mode of action, possible beneficial use and their effective utilization as sustainability enhancers. Microorganisms can be beneficially utilized in agriculture for improving soil fertility. The soil fertility enhancement can be brought about by nutrient addition (example: BNF), nutrient solubilization (Ex: PSB) or through nutrient mobilization (ex: VAM). In addition to these, many microorganisms secrete plant growth-promoting substances in the rhizospheric region that can enhance plant growth. Many microorganisms also help in suppressing pathogenic microorganisms and thus help in suppressing or minimizing disease incidence. Many microorganisms have also been found to impart stress tolerance through different mechanisms. Considering the wide range of benefits the microorganisms offer in an agroecosystem, their isolation and culture and their use as bioinoculants helps in attaining a sustainable and climate-smart agriculture production system.

## Figures and Tables

**Table 1:** Inoculation of microorganisms facilitated crop growth by the availability of nutrients.

Microorganisms	Crop Performance over Control (Untreated)	Associated Crop	References
*Glomus faciculatum Bacillus megaterium*	30% increase in yield	Banana	[74]
*Bacillus firlmus Enterobacter* spp. *Burkholderia* spp. *Pseudomonas* sp. *Paenibacillus kribbensis*	12% enhancement in yield, increase in the tillers number and yield	Paddy	[74]
*Phosphobacterium variety SBS 1*	Increase in yield up to 70%	Sword bean	[74]
*Azotobacter chroococcum*	Increase yield up to 74%	Wheat	[75]
*Rhizobium lupini*	Higher Nitrogen shoot content, enhanced nutrient uptake, increased root and shoot weight	Alfalfa	[76]
*Pseudomonas cepacia R85, Pseudomonas fluorescens R22*	Increased plant height and yield	Wheat	[77]
*R. phaseoli*	Increase in root nodule formation	Clover	[78]
*Frateuria aurantia* isolate KSBD-58	Growth parameters, head diameter, test weight, seed yield and potassium content	Sunflower	[79]
Co-inoculation of *Rhizobium meliloti, Paenibacillus polymyxa* and *Bacillus megaterium*	Increase in dry matter, nodule and dry root weight	Bean	[80]
*Bradyrhizobium japonicum*	Enhanced root nodule formation and yield	Soybean	[81]
*Azospirillum brasilense, Bacillus*, and *Pseudomonas fluorescens*	Shoot yield, P accumulation in cane, reduced P fertilization by 75%	Sugarcane	[82]
*Rhizobium leguminosarum* sv. *viciae*	Enhanced nodulation and growth	Pea	[83]
*Azospirillum brasilense*	Increased leaf chlorophyll index, stem girth, grain yield	Maize	[84]
*Azospirillum brasilense*	Seed inoculation increased growth and grain yield (26.7%), nutrient uptake	Wheat	[84,85]
*Azospirillum* sp.	Increase of growth and yield	Finger millet	[23]
*Azospirillum* brasilense Ab-V5	Increased growth, yield and nitrogen use efficiency (NUE)	Maize	[86]
Acinetobacter sp. RC04 and Sinorhizobium sp. RC02	Seed germination, seedling growth	Safflower	[86]
Co-inoculation of *BradyrhizobiumAzospirillum, Bacillus and Pseudomonas*	Increased nodule number (11.40%) and biomass of nodule (6.47%), root (12.84%), and shoot (6.53%)	Soybean	[87]

**Table 2:** Growth hormone-producing microbes.

Phytohormone/ACC Deaminase	PGP Bacteria	References
ACC deaminase	*Arthrobacter*, *Streptomyces* spp., *Leifsonia soli* sp. nov, *Microbacteriumazadirachtae* sp. nov., *Rhodococcus* sp. R04, *Micrococcus* spp.,	[185,186]
Auxin/IAA	*Actinomyces* sp., *Bradyrhizobium*, *Bacillus megaterium*, *Frankia* sp., *Micrococcus*, *Methylobacterium oryzae*, *Nocardia* sp., *Rhizobium*, *Streptomyces* spp., *S. atrovirens*, *S. griseoviridis* K61, *S. lydicus* WYEC108, *S. olivaceoviridis*, *S. rimosus*, *S. rochei*, *S.viridis*	[187,188,189,190,191,192,193,194,195,196,197,198,199]
Cytokinins	*Arthrobacter, Frankia* sp., *Leifsonia soli*, *Rhodococcusfascians*, *Pseudomonas*, *Streptomyces* *turgidiscabies*	[186,200]
Gibberellin	*Actinomyces* sp., *Bacillus*, *Arthrobacter*, *Micrococcus*, *Nocardia* sp., *Streptomyces* sp.	[187,188,193,201,202,203,204,205,206]

**Table 4:** Antagonistic actinomycetes suppressing plant pathogens.

Diseases	Pathogen	Antagonistic Strain	References
Crown rot	*P. aphanidermatum*	*M. chalcea*	[237]
Damping-off	*P. aphanidermatum, P. aphanidermatum, F. oxysporum*	*A. campanulatus, S.* *rubrolavendulae* S4, *Streptomyces* sp.	[237,238,239]
Lupin root rot	*P. tabacinum,* *F. oxysporum,* *R. solani*	*A. missouriensis,* *S. halstedii* AJ-7, *S. vinaceusdrappus*	[240,241]
Root rot of lupine	*P. cinnamomi*	*M. carbonacea*	[240]
Root rot of turfgrass	*P. infestans*	*S. violaceusniger* strain YCED-9	[242]
Wood rot	*P. chrysosporium,* *P. placenta,* *C. versicolor,* *G. trabeum*	*S. violaceusniger* XL-2	[243,244]
